# Trends in Incidence of End-Stage Renal Disease Among Persons With Diagnosed Diabetes — Puerto Rico, 1996–2010

**Published:** 2014-03-07

**Authors:** Nilka Ríos Burrows, Israel Hora, Desmond E Williams, Linda S Geiss

**Affiliations:** 1 Division of Diabetes Translation, National Center for Chronic Disease Prevention and Health Promotion, CDC

During 2010, approximately 6,091 persons aged ≥18 years in Puerto Rico were living with end-stage renal disease (ESRD) (i.e., kidney failure that requires regular dialysis or kidney transplantation for survival). This included 1,462 persons who began treatment for ESRD in 2010 (1). Diabetes is the leading cause of ESRD in Puerto Rico, accounting for 66% of new cases in adults, followed by hypertension, which accounts for 15% of the cases (1). Although the number of adults initiating ESRD treatment (i.e., dialysis or kidney transplantation) in Puerto Rico each year who have diabetes listed as a primary cause (ESRD-D) has increased since 1996 (1,2), ESRD-D incidence among adults with diagnosed diabetes has not shown a consistent trend (2). To assess recent trends in ESRD-D incidence among adults aged ≥18 years in Puerto Rico with diagnosed diabetes and to further examine trends by age group and sex, CDC analyzed 1996–2010 data from the U.S. Renal Data System (USRDS) and the Behavioral Risk Factor Surveillance System (BRFSS). After increasing in the late 1990s, ESRD-D incidence decreased during the 2000s among adult men and among persons aged 18–44 years with diagnosed diabetes in Puerto Rico. Throughout the period, ESRD-D incidence among adult women and among persons aged 45–64 and ≥75 years with diagnosed diabetes did not show a consistent trend, and ESRD-D incidence among persons aged 65–74 years with diagnosed diabetes increased. Increased awareness of the risk factors for kidney disease and implementation of effective interventions to prevent or delay kidney disease among persons with diagnosed diabetes might decrease ESRD incidence in Puerto Rico, particularly among women and older persons.

USRDS collects, analyzes, and distributes ESRD clinical and claims data to the Centers for Medicare and Medicaid Services (CMS) (3). Health-care providers are required by law to complete the CMS Medical Evidence Report for each new patient with ESRD. USRDS collects demographic and ESRD-related information (e.g., date patient was first treated and diagnosed primary cause of kidney failure). Throughout the period, in Puerto Rico, the proportion of new ESRD cases that were ESRD-D ranged from 58% to 67% (1). ESRD-D incidence per 100,000 persons with diagnosed diabetes was calculated by dividing the number of adults aged ≥18 years with a new diagnosis of ESRD-D (determined by their initiation of treatment) by the estimated number of adults aged ≥18 years with diagnosed diabetes. The USRDS Renal Data Extraction and Referencing System, an online data querying application (1), was used to determine the number of adults aged ≥18 years in Puerto Rico initiating ESRD treatment with diabetes listed as a primary cause for each year during 1996–2010. The number of adults aged ≥18 years in Puerto Rico with diagnosed diabetes was estimated from the BRFSS, which conducts state-based, random-digit—dialed telephone surveys in the 50 states, the District of Columbia, Puerto Rico, and other U.S. territories. During 1996–2010, BRFSS response rates for Puerto Rico ranged from 65% to 89%. BRFSS respondents were classified as having diagnosed diabetes if they answered “yes” to the question, “Has a doctor ever told you that you have diabetes?” Women who were told that they had diabetes only during pregnancy were classified as not having diabetes. BRFSS data were weighted to represent the noninstitutionalized population in Puerto Rico.

ESRD-D incidence rates were calculated for the adult population with diabetes overall, by age group, and by sex, and rates were age-adjusted by the direct method to the 2000 U.S. standard population. Trends were analyzed using joinpoint regression, which uses permutation tests to identify points (i.e., joinpoints) where linear trends change significantly in direction or magnitude (e.g., zero joinpoints indicate a straight line). The rate of change for each trend is tested to determine whether it is significantly different from zero, and each trend in the final model is described by an annual percentage change (APC) with a 95% confidence interval. Results were considered significant if p<0.05.

During 1996–2010, the total number of adults aged ≥18 years in Puerto Rico who began ESRD-D treatment each year increased from 536 to 970. During the study period, the age-adjusted ESRD-D incidence rates in Puerto Rico increased significantly, from 152.8 per 100,000 population with diabetes in 1996 to 230.8 in 2000 (APC = 12.4%; p=0.01), and then declined to 203.1 in 2010 (APC = −2.3%; p=0.02) (Figure 1, Table). Among men, the age-adjusted ESRD-D rates increased from 171.9 per 100,000 population with diabetes in 1996 to 371.3 in 2001 (APC = 13.4%; p<0.001), and then declined to 279.8 in 2010 (APC = −3.1%; p=0.03). Among women, however, age-adjusted rates showed no consistent trend. Rates were lower for women than men throughout the period. Among persons aged 18–44 years, ESRD-D rates increased from 96.4 per 100,000 population with diabetes in 1996 to 201.6 in 2002 (APC = 13.1%; p=0.004), and then declined to 132.0 in 2010 (APC = −6.3%; p=0.01) (Figure 2, Table). Throughout the period, rates for those aged 45–64 years and ≥75 years showed no consistent trend, and among those aged 65–74 years, rates increased, from 234.0 per 100,000 population with diabetes in 1996 to 382.0 in 2010) (APC = 2.9%; p=0.001).

## Editorial Note

ESRD is a costly and disabling condition that can result in premature death (3). Diabetes is a major risk factor for ESRD, accounting for about two thirds of new cases in Puerto Rico. During 1996–2010, the number of ESRD-D cases in Puerto Rico increased, as did the number of persons with diagnosed diabetes (2,4). After increasing in the late 1990s in Puerto Rico, ESRD-D rates decreased in the 2000s among those aged 18–44 years and among men with diagnosed diabetes. However, these encouraging trends were not found for women or for persons aged ≥45 years with diagnosed diabetes, who showed little change, except for persons aged 65–74 years, whose rates increased throughout the period.

In contrast with the ESRD-D trends in Puerto Rico, ESRD-D incidence in the U.S. population with diabetes declined during the 2000s in all age groups, in men, in women, and in Hispanics (2,5). Reasons for this decline in ESRD-D incidence cannot be determined from surveillance data but might include reductions in ESRD risk factors (e.g., hyperglycemia and hypertension) or better treatment of kidney disease among persons with diagnosed diabetes. Why trends were not as encouraging in the population with diabetes in Puerto Rico is unknown; a particular concern is the increasing trend in incidence among those aged 65–74 years. Additional strategies might be needed to reduce ESRD risk factors among persons with diabetes aged ≥45 years and among women. However, reducing ESRD-D incidence among persons aged 65–74 years likely will be challenging because persons with diabetes are surviving longer and ESRD typically occurs 10–20 years after diabetes onset (6). Furthermore, the number of new ESRD-D cases is likely to continue to increase as the population ages and the number of persons with diabetes increases (2,4).

What is already known on this topic?

Diabetes is the leading cause of end-stage renal disease (ESRD) in the United States. In the 2000s, the incidence of ESRD attributed to diabetes (ESRD-D) in the U.S. total and U.S. Hispanic populations with diagnosed diabetes declined.

What is added by this report?

After increasing in the late 1990s, ESRD-D incidence among adults in Puerto Rico with diagnosed diabetes decreased in the 2000s in men and in persons aged 18–44 years. From 1996 to 2010, ESRD-D incidence among adults in Puerto Rico with diagnosed diabetes did not show a consistent trend among women and among persons aged 45–64 years and ≥75 years, and it increased among persons aged 65–74 years.

What are the implications for public health practice?

Further research might be considered to learn why ESRD-D incidence trends in the population with diabetes were not as encouraging in Puerto Rico as in the United States, and especially why ESRD-D incidence is increasing among persons aged 65–74 years. Additional strategies might be needed to reduce ESRD risk factors among persons aged ≥45 years and among women with diagnosed diabetes.

The findings in this report are subject to at least three limitations. First, data were collected for patients whose ESRD treatment was reported to CMS and do not include patients who died before receiving treatment or persons who refused treatment. Second, changes in ESRD-D incidence might have been caused by factors other than a true change in disease incidence. These factors might include access to or acceptance of ESRD treatment, changes in treatment and care practices, or changes in physician reporting of the primary cause of kidney failure. Furthermore, revised diagnostic criteria for diabetes in 1997 might have led to a greater number of persons being detected with diabetes earlier in the disease process (7) who have not had diabetes long enough to develop ESRD, thus possibly lowering ESRD-D rates. Finally, BRFSS data during the study period were limited to adults living in noninstitutional households who had landline telephones. These sample restrictions and lower response rates (65% in 2000) might have biased the estimated population with diagnosed diabetes.*

Continued interventions, such as blood glucose and blood pressure control (8,9), to improve diabetes care and to increase awareness of risk factors for kidney disease in persons with diabetes might be considered to reduce ESRD incidence in Puerto Rico, particularly among women and among older persons. Diabetes prevalence estimates by Puerto Rico municipio (equivalent to a county or township) might assist public health officials in targeting interventions for promoting kidney health (4). To assess progress, CDC’s National Diabetes Surveillance System monitors ESRD-D incidence trends in Puerto Rico (2). Ultimately, prevention of type 2 diabetes and improved diabetes management are likely to contribute in part to the prevention of kidney disease and ESRD (8,9). CDC works with state and territorial health departments diabetes prevention and control programs and other public and private partners to reduce the incidence of type 2 diabetes and to improve outcomes for persons with diabetes. CDC’s National Diabetes Prevention Program† supports the implementation of community-based lifestyle programs throughout the United States and Puerto Rico for persons at high risk for type 2 diabetes. The National Diabetes Education Program,§ sponsored by CDC and the National Institutes of Health (NIH), develops and disseminates materials and resources in Spanish to educate persons about diabetes prevention and control. Likewise, NIH’s National Kidney Disease Education Program¶ promotes kidney disease awareness in the Hispanic population.

## Figures and Tables

**FIGURE 1:**
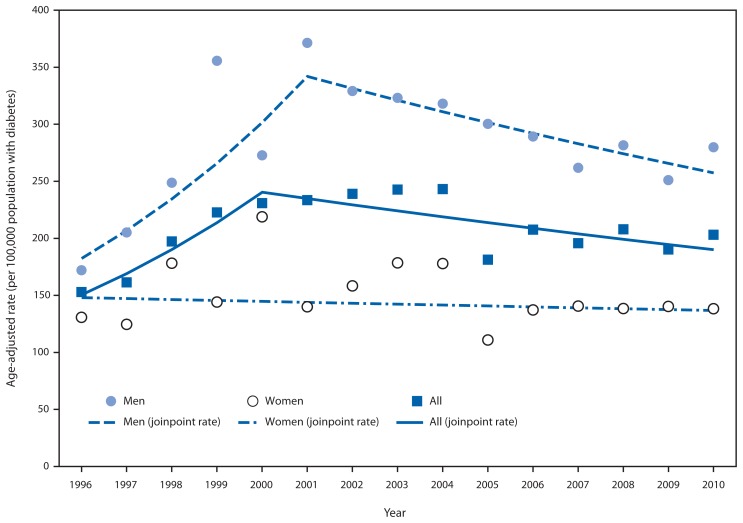
Age-adjusted* rates^†^ (per 100,000 population with diabetes) of adults aged ≥18 years initiating treatment for end-stage renal disease attributed to diabetes, by sex — Puerto Rico, 1996–2010 * Based on the 2000 U.S. standard population. ^†^ Observed rates and modeled rates using joinpoint regression.

**FIGURE 2:**
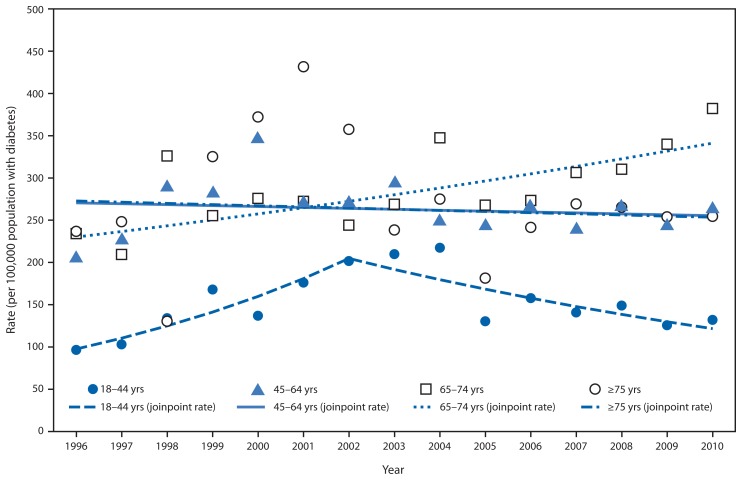
Rate (per 100,000 population with diabetes)* of adults aged ≥18 years initiating treatment for end-stage renal disease attributed to diabetes, by age group — Puerto Rico, 1996–2010 * Observed rates and modeled rates using joinpoint regression.

**TABLE:** Rate (per 100,000 population with diabetes) of adults aged ≥18 years initiating treatment for end-stage renal disease attributed to diabetes, by age group and sex, and trend analysis, by period — Puerto Rico, 1996–2010

	Rate*		Trend analysis							
		
	1996	2010	Period 1	APC	(95% CI)	p-value	Period 2/3	APC	(95% CI)	p-value
**Total**										
Crude	193.5	267.9	1996–2000	11.5	(7.2–15.9)	<0.001	2000–2005	−3.9	(−7.0– −0.7)	0.02
							2005–2010	1.9	(0.1–3.7)	0.05
Age-adjusted†	152.8	203.1	1996–2000	12.4	(3.3–22.4)	0.01	2000–2010	−2.3	(−4.1– −0.5)	0.02
**Age group (yrs)**										
18–44	96.4	132.0	1996–2002	13.1	(5.1–21.8)	0.004	2002–2010	−6.3	(−10.5– −1.9)	0.01
45–64	205.9	261.9	1996–2010	−0.4	(−1.8–1.0)	0.55				
65–74	234.0	382.0	1996–2010	2.9	(1.4–4.4)	0.001				
≥75	236.8	254.5	1996–2010	−0.5	(−3.7–2.8)	0.73				
**Sex** †										
Men	171.9	279.8	1996–2001	13.4	(6.9–20.3)	<0.001	2001–2010	−3.1	(−5.7– −0.5)	0.03
Women	130.7	138.3	1996–2010	−0.6	(−2.6–1.5)	0.56				

**Abbreviations:** APC = annual percentage change; CI = confidence interval.

* Per 100,000 population with diabetes.

† Based on the 2000 U.S. standard population.

## Footnotes

* Additional information available at http://www.cdc.gov/brfss/data_documentation/index.htm.

† Additional information available at http://www.cdc.gov/diabetes/prevention.

§ Additional information available at http://ndep.nih.gov.

¶ Additional information available at http://nkdep.nih.gov/inicio.shtml.
